# Application of Artificial Intelligence Techniques for Monkeypox: A Systematic Review

**DOI:** 10.3390/diagnostics13050824

**Published:** 2023-02-21

**Authors:** Krishnaraj Chadaga, Srikanth Prabhu, Niranjana Sampathila, Sumith Nireshwalya, Swathi S. Katta, Ru-San Tan, U. Rajendra Acharya

**Affiliations:** 1 Department of Computer Science and Engineering, Manipal Institute of Technology, Manipal Academy of Higher Education, Manipal 576104, India; 2 Department of Biomedical Engineering, Manipal Institute of Technology, Manipal Academy of Higher Education, Manipal 576104, India; 3 Department of Information and Communication Technology, Manipal Institute of Technology, Manipal Academy of Higher Education, Manipal 576104, India; 4 Manipal Institute of Management, Manipal Academy of Higher Education, Manipal 576104, India; 5 Department of Cardiology, National Heart Centre Singapore, Singapore 168752, Singapore; 6 Duke-NUS Medical School, Singapore 169857, Singapore; 7 Ngee Ann Polytechnic, Department of Electronics and Computer Engineering, Singapore 599489, Singapore; 8 Department of Biomedical Engineering, School of Science and Technology, SUSS University, Singapore 599494, Singapore; 9 Department of Biomedical Informatics and Medical Engineering, Asia University, Taichung 40444, Taiwan

**Keywords:** monkeypox, mpox, artificial intelligence, machine learning, deep learning

## Abstract

Monkeypox or Mpox is an infectious virus predominantly found in Africa. It has spread to many countries since its latest outbreak. Symptoms such as headaches, chills, and fever are observed in humans. Lumps and rashes also appear on the skin (similar to smallpox, measles, and chickenpox). Many artificial intelligence (AI) models have been developed for accurate and early diagnosis. In this work, we systematically reviewed recent studies that used AI for mpox-related research. After a literature search, 34 studies fulfilling prespecified criteria were selected with the following subject categories: diagnostic testing of mpox, epidemiological modeling of mpox infection spread, drug and vaccine discovery, and media risk management. In the beginning, mpox detection using AI and various modalities was described. Other applications of ML and DL in mitigating mpox were categorized later. The various machine and deep learning algorithms used in the studies and their performance were discussed. We believe that a state-of-the-art review will be a valuable resource for researchers and data scientists in developing measures to counter the mpox virus and its spread.

## 1. Introduction

**Figure 1:**
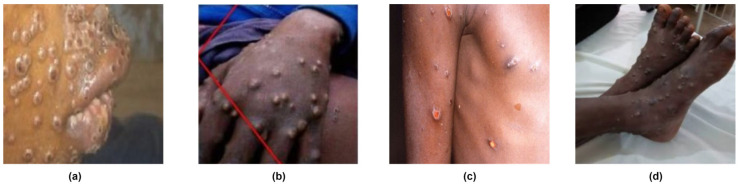
Mpox rash in the face (**a**), hand (**b**), shoulder (**c**), and feet (**d**) [7].

Monkeypox or Mpox disease, a smallpox-like illness caused by the mpox virus [1], originated primarily in the rainforests of Central and West Africa [2]. However, since the recent outbreak in May 2022 [3], it has spread to many countries, threatening to be a global epidemic. The virus is transmitted by contact with bodily fluids and respiratory droplets [4]; infected persons typically manifest symptoms for a few weeks, which include fever, swollen lymph nodes, and body rash (Figure 1). The disease is self-limiting in most affected individuals and requires only symptomatic management. However, severe medical complications can occur in some patients [5], which leads to fatality in 3–5% of cases [6]. Currently, no specific drug targets the mpox virus. Instead, therapies developed for treating smallpox in adults, including antivirals and vaccinia immune globin, are used to manage severe mpox infection [5].

The emergence of mpox amid the devastating coronavirus disease 2019 pandemic [8,9] has triggered a global alert and galvanized efforts toward a scientific reappraisal of the mpox virus. Machine learning (ML) is a subdomain of artificial intelligence (AI) and has significantly altered the healthcare infrastructure. ML and deep learning (DL) algorithms can be used on the acquired data to identify previously hidden information, monitor the patient’s health to detect, and alert about life-threatening conditions [8]. They employ scientific and mathematical methodologies for generating new information from the data. This helps to obtain accurate and robust diagnosis systems. X-rays, magnetic resonance imaging, computed tomography, and other modalities generate high-resolution images, which can sometimes be challenging to understand, even for an experienced radiologist. ML has already demonstrated that it can be highly beneficial for pathologists and radiologists as it always yields highly accurate and efficient diagnosis models [9]. Such automated systems have been used for cardiovascular abnormalities [10], fractures [11], neurological diseases [12], cancers [13], and many more [14]. ML and DL models are also used in drug and vaccine design.

The pathogenesis, etiology, transmission, clinical features, diagnosis, and management of mpox infection have been comprehensively reviewed recently [15]. Other authors focused on the science of mpox disease prevention and treatment [15,16,17], including candidate antiviral drugs [15] and immunological strategies [17]. Artificial intelligence (AI)-based approaches are increasingly harnessed to amplify research efficiency to develop countermeasures against mpox. Patel et al. [18] reviewed various AI approaches employed for detecting mpox virus, viral genome characterization, monitoring of spread, and prognosticating mortality risk. Gul et al. [19] reviewed various diagnostic methods for mpox, including image recognition, immunodiagnostics, nucleic acid, and whole-particle detection. Our article reviewed various AI applications for mpox diagnosis, forecasting, drug discovery, and other use cases.

**Figure 2:**
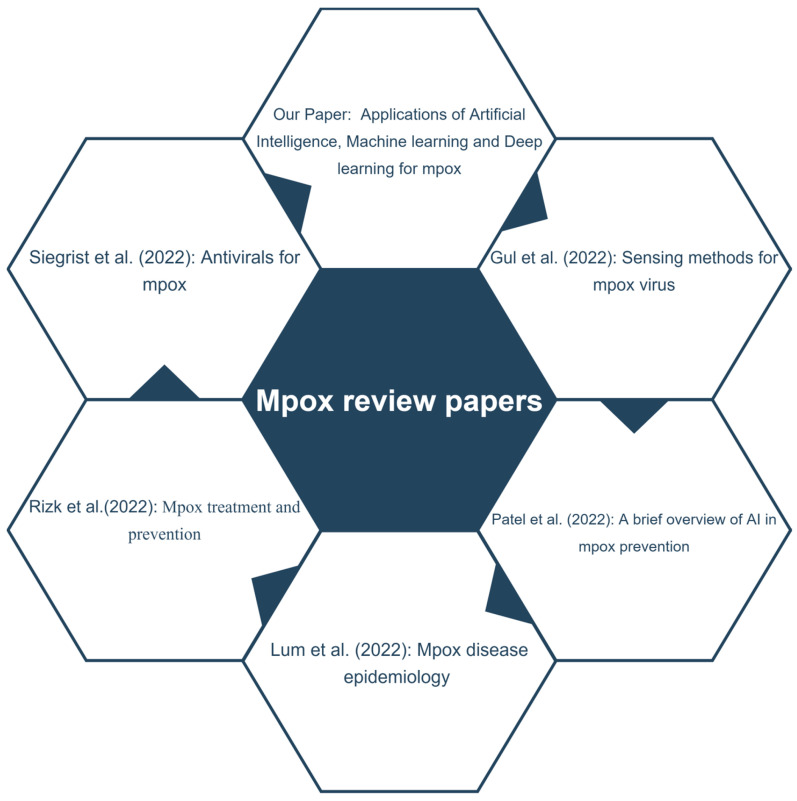
Illustration of comparison with existing review papers published on mpox detection. Siegrist et al. [16], Rizk et al. [17], Lum et al. [18], Patel et al. [19] and Gul et al. [20].

Figure 2 shows a summary of reviews published on AI for mpox-related applications, but they were not systemic reviews. Our study is a systematic study focusing on the application of applying machine learning (ML) and deep learning (DL) techniques for the automated detection of mpox. We believe a state-of-the-art review will be a valuable resource for researchers and data scientists in developing measures to counter the mpox virus and its spread. The contributions of our systematic review are as follows:

- Various diagnostics techniques using machine learning and deep learning for mpox detection are systematically reviewed.
- Articles that used AI to discover new vaccines and drugs for mpox are also reviewed.
- Other AI studies, such as epidemiological modeling of mpox infection spread and web management of information on mpox, are also included.
- A thorough discussion regarding the above applications for mpox is provided.
- Challenges and directions for future mpox research using AI are also discussed.

Due to the sudden advent of rampant infections, such as COVID-19 and Mpox, quick and rapid identification of patients without contact with hospital personnel is required [20]. Hence, various automated diagnostic strategies for mpox viruses are compiled in this review. These detection techniques can prevent the transmission of mpox by avoiding contact between patients and healthcare workers. Furthermore, several drugs and vaccines are being created to combat the virus [21]. Hence, we also reviewed various articles which used AI for drug and vaccine development. Other applications of AI, such as forecasting mpox cases and Twitter sentiment analysis, are also included in this review.

**Figure 3:**
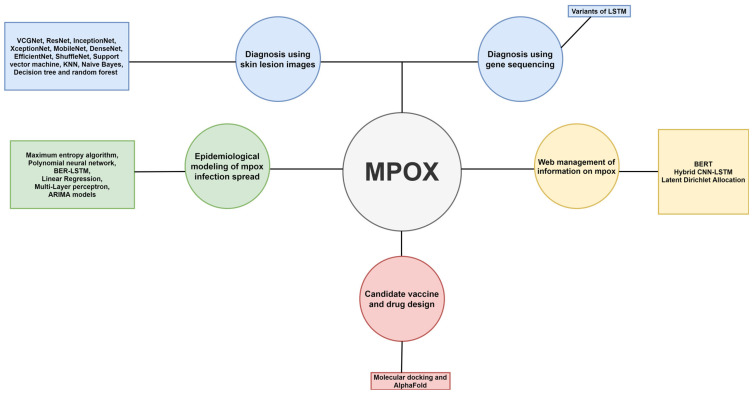
Overview of the structure of this review article.

The remainder of the article is structured as follows. Section 2 contains the review methodology. AI applications in mpox diagnosis, which comprises gene/proteomic analyses, image recognition, and clinical data-mining approaches, are presented in Section 3. Section 4 provides other AI applications, vaccine/drug discovery, forecasting of mpox cases, and countering of social engineering. The results are discussed in Section 5. Section 6 describes the limitations and future trends. Lastly, the paper concludes in Section 7. The structure of the article is described in Figure 3.

## 2. Review Methodology

**Figure 4:**
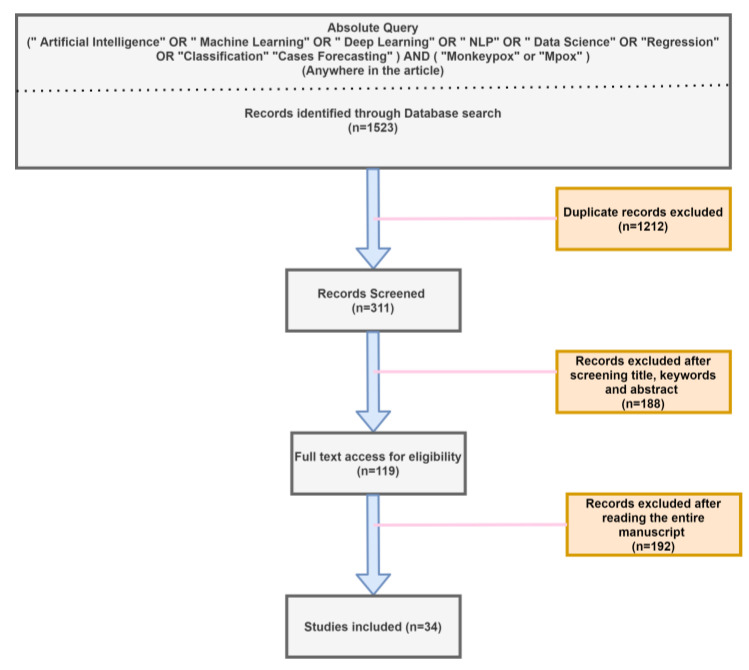
Article selection procedure using PRISMA methodology.

**Figure 5:**
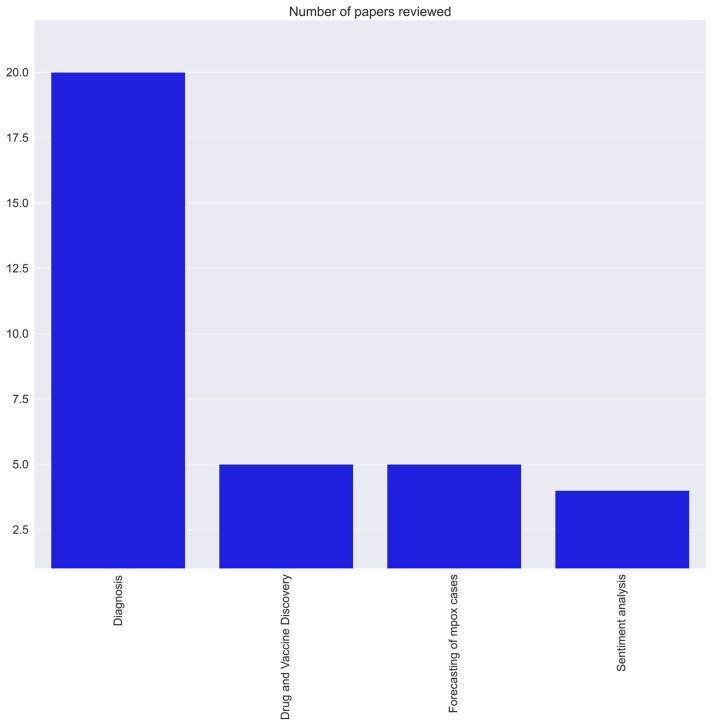
Articles selected in various categories related to mpox and AI.

A systematic literature review gathers and summarizes several research findings to analyze the work conducted by others by following Preferred Reporting Items for Systematic Reviews and Meta-Analyses (PRISMA) guidelines [22]. It involves steps such as identifying the related literature, synthesizing the findings, tabulating similar studies, and drawing various conclusions from the research. Using PRISMA methodology [22], we searched for all full-text English language articles related to AI, ML, and DL applications in human mpox research published up to 1 January 2023 in standard databases (Scopus, Google Scholar, Web of Science, PubMed, Medline, Crossref, Arxiv, and Embase) and archives of publishers (Elsevier, Springer Nature, IEEE, MDPI, Taylor and Francis, Wiley, and Arxix). We used the keyword strings “artificial intelligence and monkeypox”, “machine learning and monkeypox”, “deep learning and monkeypox”, “natural language processing and monkeypox”, “data science and monkeypox”, “regression analysis and monkeypox”, “monkeypox cases forecasting”, and their permutations; we also included substitutions of the term “monkeypox” by “monkeypox” and “mpox”. All preselected articles were screened by the first author (K.C.), who excluded animal research, case reports, letters to editors, commentaries, short reports, methods papers without results, and articles that were assessed to not actually employ AI, ML, or DL methods. A total of 1523 articles were preselected, later pruned to 34 articles after eliminating duplicate works, filtering by article titles, keywords, and abstracts, and rejection of full-text content unrelated to AI, ML, or DL (Figure 4). Since the mpox outbreak is relatively new and has lasted for a short period, only a few articles have been published. The final selection comprised 20, five, five, and four articles on mpox diagnosis, drug/vaccine discovery, forecasting of mpox cases, and sentiment analysis, respectively (Figure 5). All the above papers were carefully chosen after reading the entire content. The number of articles that included mpox detection using AI were comparatively higher than other applications.

## 3. Artificial Intelligence in Mpox Diagnosis

Polymerase chain reaction amplification of viral genetic material from skin vesicular fluid samples can provide diagnostic confirmation of mpox infection. Nevertheless, manual analysis of the genetic sequence readouts requires expertise and is time-consuming. In [23], deoxyribonucleic acid sequences of mpox and human papilloma viruses (*n* = 55 each), which manifest similar skin lesions, were numerically encoded and standardized to equal lengths using zero padding before being input into a bidirectional long short-term memory-based model for binary classification. After optimizing model parameters by trial and error, accuracy, precision, recall, and F1-score of 99.5%, 100%, 100%, and 99.95%, respectively, were attained. In [24], the authors studied the plasma proteome of a group of patients with clinical symptoms suggestive of mpox infection. There was some overlap of protein biomarkers with coronavirus infection 2019, but differences also existed that offered diagnostic clues to mpox infection. 

**Figure 6:**
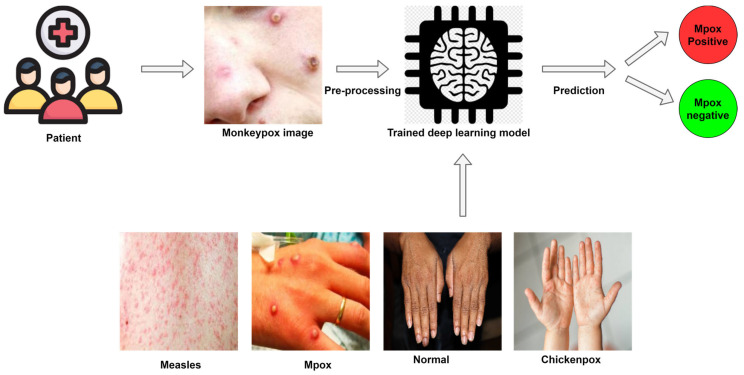
Deep learning-based system which can be deployed to diagnose mpox virus.

In the absence of laboratory polymerase chain reaction-based examination, mpox infection is clinically diagnosed by expert examination of the characteristic skin lesions. Many researchers have published the use of AI to facilitate real-time image recognition of mpox skin lesions (Table 1) (Figure 6). To address the dearth of publicly available training image datasets for mpox research, Ahsan et al. [8] built a dataset named “Monkeypox2022”, comprising images of skin lesions caused by infection with mpox, chickenpox, and measles viruses, as well as normal controls, which were collated from multiple open-source online portals. Applying transfer learning to “Monkeypox2022”, the authors used a pretrained modified VGG-16, a type of convolutional neural network (CNN), to classify mpox from other classes, attaining accuracy rates ranging from 78% to 97%. Similarly, Ali et al. [25] created the “Monkeypox skin lesion dataset”, containing images of skin lesions in mpox, chickenpox, and measles infections. They used data augmentation to increase the sample size. Among four models―InceptionV3, ResNet50, VGG-16, and an ensemble of all three―ResNet50 outperformed the rest, attaining average accuracy of 82.96%. Abdelhamid et al. [26] developed an image classification algorithm, “AI-Biruni-Earth-Radius”. Using GoogLeNet deep neural network to extract features, they attained a maximum accuracy of 98.8% for mpox detection in a multiclass dataset. In [27], among multiple classifiers used to distinguish mpox from other skin lesions, the naïve Bayes algorithm attained optimal 91% accuracy, outperforming other CNNs and shallow classifiers: GoogLeNet, VGG-16, AlexNet, k-nearest neighbor, support vector machine, random forest, and decision tree. Sitaula et al. [28] compared 13 different pretrained models with the help of transfer learning on a mpox dataset. They developed a CNN-based model to classify the skin lesion into eight disease classes and compared their solution with the help of the VGG-16 pretrained models. The accuracy, average precision, recall, and F1-score obtained for classifying mpox images were 87%, 85%, 85%, and 85%, respectively. Sahin et al. [29] developed a mobile application to detect mpox from video images of skin lesions captured and uploaded using Android phones. The CNN-based model, which was trained and tested using Matlab software embedded with TensorFlow and TensorFlow Lite, attained the best accuracy of 91.11%. In [30], the authors studied seven advanced transfer learning models for classifying 804 digitized skin images obtained from cases of mpox, smallpox, chickenpox, cowpox, and measles, as well as healthy subjects. They reported a mean precision of 85% but a poor recall of 58%. It was observed that deep learning models tend to overfit or underfit, which may be attributed to the tradeoff between training sample size and the number of trainable model parameters. In [31], various CNN-based models such as ResNet50, MobileNet-V2, EfficientNet-B0, and VGG-19, as well as an ensemble of these models were trained on images of mpox skin lesions, other rashes, and normal subjects. The ensemble model attained the best accuracy of 98.33%. 

Explainable AI (XAI) offers insights into diagnostic model behavior and performance that enhance the interpretation [32], which can potentially garner a better clinical understanding. For example, Sizikova et al. [33] used DL based on non-negative matrix factorization mutation to classify tuberculosis and mpox skin images. They incorporated XAI in the form of automatic concept discovery techniques, which made the model more accessible and interpretable. A maximum accuracy of 88.64% was obtained. XAI techniques were used in [34] to diagnose mpox on skin lesion images. A total of 572 images and 12 different models were studied. The MobileNetV2 model obtained a maximum accuracy of 98.25% among all the classifiers. Ahsan et al. [35] studied six deep learning models and obtained the best accuracy of 93% and 99% for MobileNetV2 and InceptionResNetV2, respectively, for discriminating mpox from normal skin images in an imbalanced dataset. The local interpretable model-agnostic explanations (LIME) method was incorporated into the model to comprehend and verify the model’s predictions. The insights obtained with LIME could facilitate refining the model and developing and evaluating other deep learning models, including those trained using imbalanced datasets. 

Alcala-Rmz et al. [36] used a CNN model for mpox diagnosis. They implemented the MiniGoogLeNet for various epochs. The dataset comprised 2062 images, of which 1439 were chickenpox and measles images, and 1168 were mpox images. A maximum accuracy of 97.08% was attained when 50 epochs were used. Khafaga et al. [37] used a deep CNN to classify mpox images. The dataset from Kaggle consisted of 293 normal, 279 mpox, 107 chickenpox, and 91 measles cases. A maximum accuracy of 98.83% was attained. Haque et al. [38] used attention mechanisms and deep learning models to classify mpox in humans. Five deep learning models were implemented, and a maximum accuracy of 84% was attained. Saleh et al. [39] used AI and data mining techniques to classify mpox. An improved binary chimp optimization was used for feature selection. The maximum accuracy, precision, and recall of 98.48%, 91.11%, and 89%, respectively, were obtained. 

The transfer of sensitive medical images raises privacy concerns. In [40], a secure data aggregation scheme was proposed to minimize cyber threats. Instead of transmitting raw data, blockchain-based data acquisition and federated learning were employed to assemble the data in the form of trained models. Using ResNet18 to perform binary classification of trained models constructed from images of skin lesions of mpox versus other conditions, the scheme attained 99.81% accuracy, which demonstrated the feasibility of fully secure and remote automated image-based mpox diagnosis. Joshua et al. [41] used a neuro-fuzzy model to detect the mpox virus. Their architecture, “MDiNFIS”, embodied both hardware and software. Leveraging the uncertainty-handling capability of fuzzy logic systems and the learning capability of artificial neural networks, they built a diagnostic system for mpox detection.

**Table 1:** Summary of published artificial intelligence-based mpox classification models using skin images.

Paper	Images (*n*)	Classifier (s)	Acc (%)	Pre (%)	Rec (%)	F1 (%)
Ahsan [8]	Mpox, chickenpox, measles, normal	VGG-16	97	97	97	97
Ali [25]	Mpox (102), others (126)	VGG-16, ResNet50, Inception-V3, ensemble	82.96	85	81	83
Abdelhamid [26]	Mpox (279), chickenpox (107), measles (91), normal (293)	AlexNet, VGG-19, GoogLeNet, ResNet50	98.8	-	63	74
Kumar [27]	-	AlexNet, GoogLeNet, VGG-16, support vector machine, k-nearest neighbor, naïve Bayes, decision tree, random forest	91.11	-	-	-
Sitaula [28]	Mpox, chickenpox, measles, normal	13 deep learning models.	87.13	85.44	85.47	85.4
Sahin [29]	Mpox (102), others (126)	Modified MobilNetV2	91.11	-	-	-
Islam [30]	Mpox, chickenpox, smallpox, cowpox, measles, normal	ResNet50, DenseNet121, Inception-V3, SqueezeNet, MobileNet-V2, ShuffleNet-V2, ensemble	83	-	58	-
Saavedra [31]	Mpox (100), other rashes (100), normal (100)	VGG-16, VGG-19, ResNet-50, MobileNet-V2, EfficientNet, ensembles	98.33	-	-	-
Sizikova [33]	Mpox, tuberculosis	VGG-16, EfficientNet-B3	88.64	-	-	-
Akin [34]	Mpox (252), others (264)	12 deep learning algorithms	98.25	-	-	98.25
Ahsan [35]	Mpox, normal	6 deep learning models	99	-	-	-
Alcalá-Rmz [36]	Mpox, control	MiniGoogLeNet	97.08	-	-	-
Khafaga [37]	Mpox, control	Al-Biruni Earth radius optimization-based stochastic fractal search	98.83	-	85	80
Haque [38]	Mpox, others (total 2142)	5 deep learning algorithms	84	90	89	90
Saleh [39]	Mpox (296), others (204)	Various machine learning and deep learning models	98.48	91.11	89	-
Islam [40]	Mpox (1428), others (1764)	ResNet18, block-chain federated learning for privacy protection	99.81	-	-	-

Acc, accuracy; F1, F1-score; Pre, precision; Rec, recall.

## 4. Other Applications of AI in Combating Mpox

### 4.1. Epidemiological Modeling of Mpox Infection Spread

Researchers have used AI models to predict mpox outbreaks (Table 2). Arotolu et al. [42] used a maximum entropy algorithm to model the environmental variables in 116 spatially unique cases of prior mpox infections from 2017 to 2021 in Nigeria. The model―the top five features being precipitation, human population density, elevation, and maximum and minimum temperature―accurately predicted (area under the curve of 92%) conditions and geographies conducive to mpox spread, facilitating resource distribution to at-risk regions in the country. Majumder et al. [43] trained a polynomial neural network on mpox case data collected between 6 May 2022 and 28 July 2022 to develop a predictive model that could forecast mpox cases developing over the next 100 days. In [44], a proposed “BER-LSTM” model based on long short-term memory (LSTM) network with the hyperparameters tuned using the “AI-Biruni Earth Radius” algorithm was proposed to predict mpox disease spread. Incorporating statistical methods prior to training, such as analysis of variance, regression, and Wilcoxon tests, the hybrid algorithm attained a low mean bias error of 0.06%. Yasmin et al. [45] developed a prediction model that used regression analysis to forecast mpox outbreak. Among nine algorithms, the model yielded the best performance with mean absolute error and root-mean-square error of 146.29 and 204.75, respectively. For forecasting mpox cases, Qureshi et al. [46] compared various time series AI models such as autoregressive integrated moving average (ARIMA), extreme machine learning, support vector machine, and multilayer perceptron. The latter was found to be the most reliable. 

### 4.2. Candidate Vaccine and Drug Design

Multi-epitope vaccines have the potential to generate specific immunogenic responses based on conserved epitopes in complete antigenic sequences [47], thus avoiding responses against unfavorable epitopes that might induce immunopathogenic or immune-modulating responses against the host. Using the Virus Pathogen Database and Analysis Resource Database [48] to retrieve mpox proteins, nine overlapping epitopes were chosen to create multi-epitope vaccination constructs associated with appropriate linkers and various adjustments to improve the immune system responses. In [49], 176 genome-encoded protein structures were screened as candidate mpox vaccines using immunoinformatics. The final model possessed excellent binding energy of 98.37% kcal/mol to the mpox virus. In [50], three extracellular antigenic proteins were studied using AI simulations of B and T lymphocyte immune response. The silico analysis was used to select the best candidate for further vaccine development against mpox. 

Altayb et al. focused on three mpox proteins that play crucial roles in viral replication; they used various in silico techniques such as molecular docking, computational modelling, and molecular dynamics simulations to digitally screen 1615 FDA-approved drugs for activity against these proteins [51]. Fludarabine, an anticancer therapeutic, was discovered to have the optimal docking score (7.53 kcal/mol) for the mpox protein. Lam et al. [52] used a DL server, AlphaFold, to define the structure of the 304-amino-acid mpox E8 protein. They discovered that diosmin and flavin adenine dinucleotide, both commercially available drugs, could potentially be repurposed to target the E8 protein, with maximum binding energies being 9.69 kcal/mol and 7.65 kcal/mol, respectively.

### 4.3. Web Management of Information on Mpox

Natural language processing (NLP) is an important tool for collating accruing web information generated on specific topic labels. Kolluri et al. [53] developed a browser extension named “POXVERIFI” to verify information about mpox on the internet. Their bidirectional encoder representations from the transformers (BERT) model attained 96% validation accuracy. As more users installed the extension and rate articles, crowd-sourced votes automatically generated accurate labels for new sources. The labeled data could then be leveraged to measure mpox-related misinformation. Mohbey et al. [54] used a hybrid CNN–LSTM model to classify mpox tweets into positive, negative, and neutral classes. Their model attained an absolute accuracy of 94% for assessing the sentiments of Twitter users on mpox. Unsupervised ML was used to analyze 352,182 (duplicate tweets and retweets were excluded) Twitter posts on mpox in [55]. The topics were clustered into three themes (stigmatization of minorities, safety concerns, and lack of faith in institutions), and transformers such as BERT and BERTopic were used to perform sentiment analysis. A total of 15,936 mpox tweets in German were analyzed in [56] using a mixed-methods research methodology and ML. The authors opined that a multidisciplinary strategy could be used to minimize and prevent mpox-related misinformation. A summary of articles related to AI mpox and web information is given in Table 3.

## 5. Discussion

Mpox emerged in early 2022 and has spread to many different countries. Polymerase chain reaction (PCR) is the gold standard for diagnosing this virus. However, PCR tests are prone to false-negative and erroneous results. They also consume substantial time and require trained medical professionals to conduct the test. In the first part of this comprehensive review, various ML and DL techniques used to diagnose mpox were screened. Most of the researchers used skin lesion images to detect the virus. Rashes generally appear on the patient skin, and these classifiers can utilize these features for accurate diagnosis. Similar diseases which cause skin rashes, such as measles, chickenpox, and smallpox, were present in the datasets. Hence, the AI algorithm diagnosis can be subjected to thorough investigation in the hospitals. The AI-based system can be deployed in various healthcare facilities to assist clinicians. This automated diagnosis can be useful in countries that lack infrastructure and medical technology. It can also be used to confirm the PCR tests. The DNA sequences were also used to diagnose mpox in one study. Most of the algorithms were able to obtain high accuracy in detecting mpox. The most widely used algorithm was CNN and its variants. Conventional ML algorithms such as support vector machine, K nearest neighbors, decision tree, and random forest were also utilized. We believe that all three methods should be used to diagnose mpox if available. Mpox detection using skin lesion images and deep learning models were highly effective since the models could easily distinguish between mpox and other similar diseases. The deep learning models can be used in parallel to the PCR tests to prevent false negative results. The most promising drugs were diosmin, flavin adenine dinucleotide, and fludarabine. Many studies used explainable artificial intelligence (XAI) for mpox detection. This enables the clinicians to understand and convince the decisions taken by the deep learning classifiers. LIME and gradient-weighted class activation map (Grad-CAM) can also be used to visualize the lesions in the images [57]. Furthermore, we plan to explore the possibility of the model’s authenticity when employed in a real-world scenario, as it can be influenced by noise. Hence, computing the uncertainty of the model will help AI specialists understand the reliability in a noisy environment [58]. 

For trustworthy deep learning detection, progress in several areas, such as software engineering, XAI, and ML ops, is required. Medical validation must be performed by doctors to establish the reliability of the models. The algorithm’s robustness must be measured, and the AI systems must be continuously audited. Cyberattacks in deep learning have also become common. Neural architectures must be protected from malicious users and threats. Model and data uncertainty issues must also be addressed. 

Furthermore, we reviewed other ML studies that can help us manage mpox. Much wrong information is being published online related to mpox, such as its infection rate and duration, symptoms, and preventive measures. NLP techniques were used to mitigate this problem. The public sentiments of users on mpox were also analyzed. The majority of the researchers used the BERT technique to perform sentiment analysis. Currently, there are no drugs or vaccines to treat mpox. Various drug designing and repurposing techniques using ML were also included in this review. Drugs such as fludarabine, diosmin, and flavin adenine dinucleotide (FAD) have shown promising results. It is important to discover new drugs to prevent severe virus symptoms. ML and DL models were also used to forecast the number of cases in various countries. LSTM was the most preferred algorithm for prediction. Models such as ARIMA and MLP were also utilized. Evaluation metrics such as mean absolute error (MAE), mean squared error (MSE), and R-squared were considered to understand the efficiency of the models.

In total, 34 published articles on mpox and AI were reviewed. This systematic literature review can help researchers from AI, computer science, data science, and medicine understand various applications that can be used for a potential mpox outbreak. 

**Figure 7:**
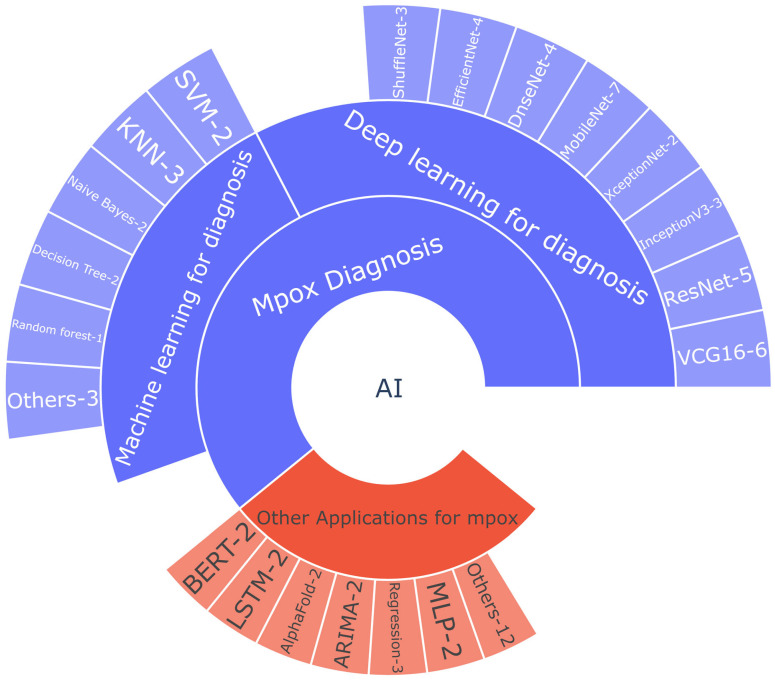
Sunburst plot of AI studies using Mpox data. The inner layers depict various categories of applications; the outer layers depict the algorithms used, with each corresponding use frequency indicated by a numbered suffix. For example, MobileNet-7 indicates that MobileNet has been used for mpox diagnosis in seven studies.

Figure 7 shows the various ML and DL algorithms in the reviewed studies for the different applications, with the use frequencies indicated. MobileNet was the most widely used DL algorithm for automated detection of mpox. Models such as VGG-16, ResNet, InceptionNet, DenseNet, and EfficientNet were also frequently used. ML classifiers such as k-nearest neighbor and support vector machine were used in a few studies. For specific applications such as mpox case forecast, drug discovery, and public sentiment analysis, models such as BERT, LSTM, ARIMA, and regression methodologies were employed. 

## 6. Limitations and Future Directions

### 6.1. Limitations

First, there is a shortage of open-source data for training AI models. Few public datasets are available for mpox, and most are small, limiting the use of data-hungry DL algorithms. AI applications require high data volume to avoid bias and overfitting. Hence, mpox data collection should be encouraged and publicly shared to facilitate research. The training of developed models on common open-source datasets will also allow a meaningful comparison of model performance. Second, data filtering is necessary to remove wrong mpox information and ensure data input fidelity into AI models. Third, sharing, especially of sensitive raw data, can potentially compromise privacy. Therefore, protecting sensitive information from malicious users and hackers is imperative. Data must be structured well for effective model training. With most of the online data being unstructured, it is hard to derive useful information despite abundant data.

Deep learning algorithms are also prone to false negative and erroneous results. Hence, deep learning algorithms can be combined with other machine learning techniques such as unsupervised learning and reinforcement learning. From the above studies, we can see that most researchers used skin lesion images to diagnose mpox. However, it is important to look into other modalities such as blood tests and laboratory markers. When multiple modalities are used, the results are generally more reliable. The strains of some viruses continue to mutate after a certain amount of time. Hence, the models must also be tested on data consisting various mutations. The models must also be tested on various datasets from different geographical territories. 

### 6.2. Future Directions

**Figure 8:**
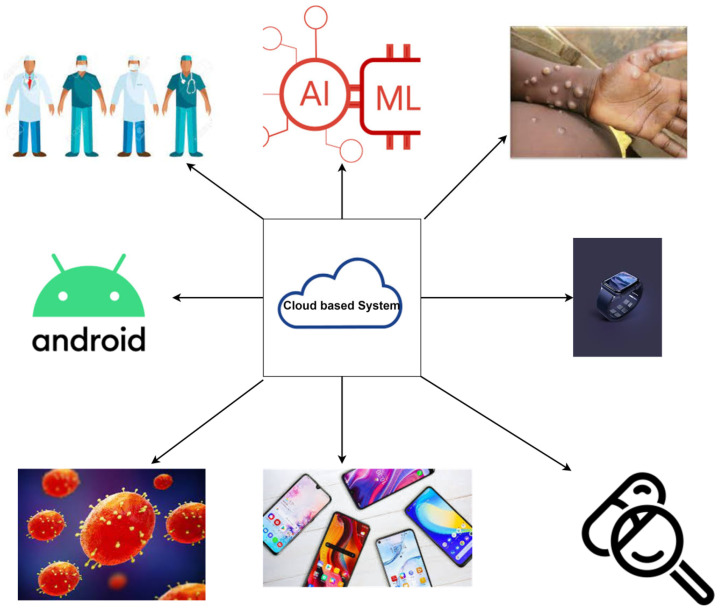
Cloud-based system for mpox management.

We see the potential for a secure cloud-based system for comprehensive mpox management (Figure 8). Patient data, wearable devices, smartphone data, and AI models can be deployed on the cloud. Doctors can access these via the cloud infrastructure, including uploaded skin lesion images for remote mpox diagnosis. Clinical, laboratory, epidemiological, demographic, and other parameters can also be incorporated to allow systems-wide analysis for healthcare resource allocation. To prevent the infection from spreading, teleconsultation via remote video conferencing can also be integrated into the system, and appropriate symptomatic treatment can be dispensed remotely for mild cases. Such a system is scalable, and most of the researchers use datasets from a single source or geographical regions. With a cloud system, data from multiple locations can be gathered and analyzed to detect trends for high-level strategic planning. Furthermore, the correlation between dataset size and classifier performance can be quantified via sensitivity analysis [59]. We may then analyze the study results to determine how much information is required and how small a dataset is needed to accurately anticipate performance on larger datasets. XAI methods (e.g., Shapley additive explanation, LIME, Eli5, and QLattice) can be incorporated to enhance the interpretability of the model outputs, which will help researchers and clinicians better understand the model predictions using different visualization techniques [32]. Computational costs of the applications can also be computed and compared.

## 7. Conclusions

The current Mpox outbreak is a cause for global concern. Although not as fatal as the coronavirus infection in 2019, it would be wise to prepare for worsening outcomes. In recent years, AI has significantly accelerated adoption in health science research and applications. Here, we comprehensively reviewed the latest AI-related methods applied to combat the Mpox virus. AI models for diagnosing Mpox, predicting Mpox outbreaks, vaccine and drug discovery, and other related applications were explored in depth. We believe that this review can help researchers and medical professionals understand the various AI applications in place, which can be further expanded or refined if the Mpox outbreak worsens. Key issues and future directions were also discussed.

## Figures and Tables

**Table 2:** Published artificial intelligence-based models for predicting the number of mpox cases.

Paper	Dataset	Model	Results
Arotolu. [42]	116 mpox patients from Nigeria	Maximum entropy algorithm	Area under curve 92%
Majumder [43]	Mpox cases from 6 May to 28 July 2022	Polynomial neural network	Predicted mpox cases would decrease after 20 October 2022
Eid [44]	Global mpox cases dataset, Kaggle	BER-LSTM	Mean absolute error 15.25
Yasmin [45]	Global mpox cases dataset, Kaggle	Nine forecasting models	Mean absolute error 146.29
Quereshi [46]	From website “Our World in Data” between 6 May to 28 July 2022	Multi-layer perceptron, ARIMA model	Mean absolute error 32.59

**Table 3:** Published artificial intelligence-based models for mpox-related web information.

Paper	Use Case	Dataset	Model	Results
Kolluri [53]	Handling mpox misinformation using a browser extension	170 actual facts and 55 wrong facts collected from the internet	BERT-based machine learning model.	96% accuracy
Mohbet [54]	Sentiment analysis of Twitter users	Twitter posts about mpox	Hybrid CNN–LSTM	94% accuracy
Ng [55]	Sentiment analysis of Twitter users	352,182 Twitter posts	BERT, BERTopic	-
Al Ahdal [56]	Sentiment analysis of Twitter users in Germany	15,936 Twitter posts from Germany	Latent Dirichlet allocation	-

## Data Availability

Data will be made available on request.
